# Measuring intellectual capital with financial data

**DOI:** 10.1371/journal.pone.0249989

**Published:** 2021-05-03

**Authors:** Carlos M. Jardon, Xavier Martinez-Cobas

**Affiliations:** 1 Department of Applied Economics-ECOBAS, University of Vigo, Vigo, Galicia, Spain; 2 National research University Higher School of Economics, Perm, Russia; 3 Department of Accounting and Financial Economics-ECOBAS, University of Vigo, Vigo, Galicia, Spain; Institute for Economic Forecasting, Romanian Academy, ROMANIA

## Abstract

Intellectual capital is defined as the set of intangible assets that generate value for the company. Normally, the models that measure the intellectual capital make use of investments in intangible assets, as indicators of the generation of value by the company; or are based on a holistic measure, using another focus to validate. This research proposes a new method to measure intellectual capital, reconciling the use of financial measures for the management of intellectual capital and its antecedents in triangulated indices; it also determines relationship path coefficients, between constructs developed from a general conceptual model, based on the academic and professional literature. The proposed method combines component indicators with holistic indicators using a structural equation model, allowing differentiating the components of intellectual capital from the stock of intellectual capital. The method is applied to more than 1,600 European companies from 2004 to 2015 to assess its validity, presenting the monetary value of intellectual capital in these companies. The results allow a comparison of the situation of intellectual capital in companies in different countries and industries, opening an opportunity to disclosure intellectual capital.

## 1. Introduction

Intellectual capital is becoming a crucial performance and long-term growth factor in a knowledge-based economy where companies identify their core competence as intangible assets rather than tangible assets [1,2].

There are numerous definitions and approaches for measuring the idea of intellectual capital [3–10].

Through the contribution of different disciplines, a significant number of measurement models have emerged [9,11–17]. However, these models do not fully describe the value of intangible assets, such as intellectual capital, making it difficult to manage [18].

From the accounting field and especially in the accounting of intellectual capital there is also a need to establish measurement models [19–21], but today we need models that add value for research in IC accounting [22].

These models have been presented with different approaches, using available information. In particular, from the accounting point of view, previous studies have tried to assess the intellectual capital from financial information [9,11,13,23–27], but their results about solutions do not agree.

Summarizing previous studies [10,12,13,19,24,28–32], two great approaches can be considered: component methods and holistic methods.

Component methods divide intellectual capital into components, measure each component, and aggregate their value [11,13,33–36].

Holistic methods evaluate the intellectual capital from the market value generated by it [9,11,33].

The component methods have been criticized because they present contradictory aspects. Users of these methods consider that the interactions of the components, contribute considerably to the value of intellectual capital [37], but these interactions are omitted in focusing on the measurement of individual components [16]. Besides, it is difficult to obtain financial information about individual components. Finally, only those aspects of the components that are measurable are included, but possibly many other aspects remain that are impossible to measure accurately [9].

The holistic method is criticized because it assumes that all the value of the company over book value, is due to the intellectual capital. Also, this method identifies the monetary value of intellectual capital with the value generated by intellectual capital and other types of capital in the company.

The proposed method combines component indicators with holistic indicators using a structural equation model. This model has not previously been used in measurement methods based on financial data. The model allows differentiating the components of intellectual capital from the stock of intellectual capital, an aspect not previously analyzed. Consequently, this method answers a great number of these criticisms.

The disclosure of intellectual capital is an open problem because many companies do not publish the information about intellectual capital, and the measurement of the intellectual capital is difficult [18,38,39]. Tyskbo (2019) [40] shows how some Swedish enterprises manage their intellectual capital, but it is not easily visible in financial reports. [41] show how intellectual capital disclosure can affect the underpricing of Initial Public Offerings (IPOs).

This paper proposes a new model of measurement of intellectual capital with public financial data, that improves the limitations of previous models. As a result, this research allows reconciling the use of financial measures for the management of intellectual capital and its antecedents in triangulated indices and determines relationship path coefficients between constructs developed from a general conceptual model, based on the academic and professional literature. Also, it assesses the relative position of the participating organizations, so that intellectual capital resources can be reallocated more effectively, and establishes a basis for online trends, standards, and intellectual capital forecasts using financial data.

The paper continues with a review of the different measurement methods of intellectual capital and the proposal for an alternative. Next, the methodology is established. Then, the model is applied to 1,693 European companies from 2004 to 2015. Finally, conclusions and some management applications and practice implications are discussed.

## 2. Theoretical framework

Intellectual capital is a construct that includes intangible assets, that create value in the company. The concept of intellectual capital is not observable, because it is intangible and cannot be captured by the senses. To measure it, it is necessary to clearly define the concept. For Molloy et al. (2011) [17], a clear definition is when a construct is described accurately in four aspects: lexical, positive semantic, negative semantic, and connotative.

A lexical definition clarifies the view of the concept and how this view is based on (or departs from) representations in the previous literature. Clarifying the meaning of the definition is useful to explain how the construct is like the constructs that are positively related (positive semantic definition), and how different constructs are negatively related (negative semantic definition). These definitions are vital to establishing the convergent and discriminant validity. Connotative definitions help to clarify and advance the research, explaining to future researchers the necessary and sufficient conditions that the construct must verify when it intervenes in certain phenomena [42].

Previous papers introduce definitions of intellectual capital, suggesting different methods to evaluate it. For example, Brooking (1996) [43] defines intellectual capital as the term given to the combined market intangible assets—intellectual property and human-centered assets—that allow the company to operate. [7] indicates that intellectual capital is intellectual material, knowledge, information, intellectual property, and experience that can be used to create wealth. It is collective brainpower or useful knowledge. [44] state that intellectual capital includes all processes and assets, that are not normally shown on the balance sheet, and all intangible assets (trademarks, patents, and trademarks) that modern accounting methods consider. It includes the sum of the knowledge of its members and the practical translation of knowledge. Finally [45], consider that the value of intellectual capital is provided by the difference between the market value and the book value.

The definitions of intellectual capital can be summarized as suggesting that intellectual capital is a set of intangible assets that generate value for the company [4,46].

### 2.1. Approaches to the valuation methods of intellectual capital

Once defined, because intellectual capital is not observable, it is appropriate to look for observable indicators that show us the concept. This definition includes two concepts, intangible and value, which are difficult to quantify.

To quantify a concept, it is necessary to define the method used to measure it. The literature suggests different methods, which possibly from the point of view of econometric estimation, can be summarized as direct and indirect methods, and the latter can use formative or reflective indicators [4,10,24,34,47,48].

Direct methods observe and measure elements whose combination defines the concept. Formative indicators are elements that cause the concept. Reflective indicators are elements resulting from the concept, reflecting its properties [3,17,25,49,50].

In the case of intellectual capital, the direct method is not usually feasible with financial data, and possibly needs a survey or an interview, because some of its elements are also unobservable, but each element can be estimated by previous methods. Consequently, three measurement methods are suggested, following these aspects: based on formative indicators (inputs), based on reflective indicators (outputs), or a combination. This classification overlaps the classification suggested by Lev, Radhakrishnan, and Evans (2016) [51], consisting of formative or input methods, direct or survey methods, and reflective or output methods.

A second aspect refers to the focus. Intellectual capital is a construct, but it can be considered for analyzing its various components or as a whole.

From the components focus, traditionally, intellectual capital is divided into three categories: human capital, structural (or organizational) capital, and relational (customer) capital [11].

Human capital refers to the set of values, attitudes, skills, and abilities of employees that can generate value for the company [3,52]. Human capital includes knowledge, experience, skills, creativity, teamwork, loyalty, training and education, problem-solving ability, attitude, loyalty, and the motivation of people [53,54]. Usually human capital is tacit knowledge [55].

Structural capital is “that knowledge that internalizes company (generating value for it) and remains in the organization even when their employees leave for their homes at night” (Roos et al. 1997, p. 42) [44]; therefore, structural capital is independent of individuals and is generally explicit knowledge [54]. Structural capital includes intellectual property (patents, licenses, trademarks), technology incorporated in the company, the organizational system, and culture [3,44,52,56].

Relational capital is the value of business relationships with individuals and organizations or is related in some way to creating value for the company [3,52,54]. This capital includes relationships with external stakeholders, networks with suppliers, distributors, trade organizations, partners, customer relationship management (image creation, loyalty, partner and investor network), and brands (attitude, preference, reputation, brand recognition) [57–60]. Usually, it combines explicit and tacit knowledge.

The components focus presents an aggregation problem because users of the method must decide how to aggregate the different components. This problem does not exist in a holistic focus. The holistic focus proposes to measure the intellectual capital by the value that intangibles generate for the company. The difficulty of this method is estimating the value of intellectual capital globally. Usually, the method identifies the intellectual capital value with the increase in the market value versus the book value.

Finally, a third aspect regards the sources of information used to measure intellectual capital. Initially, we could divide them into two large blocks, that condition the methods used: public and private information sources. This division is essential when analyzing the problem of revealing the intellectual capital of the company [19] because such disclosure must be made through public sources [18].

Public sources include data found in the reports of the companies in the commercial register, stock information, and data available from different sources of economic and financial information. Private sources are usually obtained by surveys, interviews, or by direct observation of the operation of the company, although there are different qualitative methods, that provide indirect information on the existence of intellectual capital [61]. In this case, the research considers the classification in financial and non-financial data.

According to this scheme, the research suggests five steps to determine the measurement method:

1. Determine the type of indicators to be used.
2. Decide the focus.
3. Select information sources.
4. Establish additional assumptions to define indicators.
5. Calculate the indicators.

Beginning with this outline, the present research revises the previous methods. Afterward, it elaborates a specific method to measure intellectual capital with financial data.

### 2.2. Measures of intellectual capital with financial data

Harrison and Sullivan (2000) [61] summarize the measures of intellectual capital into two main groups: qualitative and quantitative data. The latter breaks down into nonmonetary and monetary measures. The interest of this study is to measure intellectual capital with financial data, so, as suggested by these authors, this study will focus only on the latter group.

Sveiby (2010) [8], summarizing previous works, suggests that the measurement methods of intellectual capital could be classified into methods of direct intellectual capital (DIC), methods of market capitalization (MCM), methods of return on assets (MROA), and scorecards methods (SCM).

Direct intellectual capital estimates the monetary value of intangible assets by identifying the assets’ various components. Once these components are identified, they can be evaluated directly, either individually or as an aggregate coefficient. Accordingly, it is a component method. The way to measure each component may vary, although it is usually based on direct surveys or financial indicators. This research uses only financial indicators.

Methods of market capitalization calculate the difference between the market capitalization of a company and its stockholders’ equity, as the value of their intellectual capital or intangible assets. It is a holistic focus. This method will be analyzed later because it uses financial information.

Methods of return on assets use average earnings before tax for a period, divided by the tangible assets of the company, compared to the average of the industry. It presupposes that the difference indicates the average annual gain of intangibles. The method presupposes that it is possible to estimate the value of intangible assets or intellectual capital, by dividing the higher profits by the average cost of capital of the company or an interest rate.

The return-on-assets methods are a comparative method within the industry if all companies in the same industry use their intangible assets to generate returns similarly, and that the difference in profitability, between the company and the industry average, is due to the company’s use of its intellectual capital. This method allows for comparison only within the industry, but not between industries, which severely limits its use. Therefore, the current study does not use these methods, although the idea of comparison will be used.

Finally, scorecards methods also consider the various components of intangible assets or intellectual capital, identified, and measured by indicators reported in scorecards or as graphs. These methods usually are not based on monetary values, so this research does not use them, although the scorecards philosophy can be considered to measure components.

Lev et al. (2016) [51] suggest three approaches of measuring organizational capital that is valid for intellectual capital: measures based on the input of intellectual capital, i.e., intangible measures or having a history of intellectual capital; measures based on output, i.e., the value of the business generated by intellectual capital; and survey-based measures, i.e., looking directly through questions for the value of intellectual capital. The approaches based on inputs or output are included in our model.

Fincham and Roslender (2003) [32] put the start of the accounting of intellectual capital in the Skandia colleagues’ model [62]. For Dumay & Guthrie, (2019) [63] the Skandia model was important as they were the first company to publish an IC Statement as part of its annual report.

In the field of financial data, [64] identifies a range of metrics to measure the business’s stock of intellectual capital as, for example, market-to-book ratios, Tobin’s *q*, and calculated intangible value.

Goebel (2015) [9] classifies approaches for measuring the value of intellectual capital into three groups, according to the sources of information used: investment-based approaches (IBAs), component-based approaches (CBAs), and holistic market-based approaches (HMBAs). According to previous classifications, the author mixes information sources (investments or market value, although both are financial) with focuses (component or holistic).

The investment-based approach is essentially a measure based on inputs, the components-based approach can make use of inputs or surveys, and the third approach is based on outputs. Except for the survey-based method, the rest of the approaches use financial information. Some of these approaches use specific assumptions. The analysis of these assumptions helps to evaluate the different alternatives proposed, to establish a measurement of intellectual capital. Table 1 summarizes these classifications.

**Table 1:** Classification of measurement approach.

		Focus			
		holistic		components	
	Sources	Financial	Nonfinancial	Financial	Nonfinancial
**Method**	**Formative**			IBA, CBA	
	**Direct**				DIC, SCM, CBA
	**Reflective**	MCM, MROA, HMBA			

Consequently, the current study selects two approaches to define the measuring model: the investment approach, associated with input models; and the holistic-based market approach, associated with output models. Next, it analyses their assumptions.

### 2.3. Assumptions associated with the valuation methods of intellectual capital

Each typology has limitations, according to the assumptions additionally included.

Different methods for measuring intellectual capital include a few basic assumptions. According to Table 1, we analyze the assumptions of components and holistic modes, answering the criticisms that have been extended to both methods. The research initializes the assumption according to the method.

The components method includes some assumptions, directly related to the generation of value, and the relationship with intangible assets.

A1: Intellectual capital is the aggregation of these components, usually human capital, structural capital, and relational capital.

This assumption suggests possible formative indicators of intellectual capital. The different levels that can be considered and the way of measuring them will probably condition the practical effectiveness of this assumption. However, theoretically, the assumption should be valid for all levels considered in global terms.

A2: The expenses associated with each component of intellectual capital increase the intellectual capital of the company.

The method identifies investment and expense. Indicators can also be considered at different levels, suggesting different indicators. In addition, investments can be in time or money, in training or specialist recruitment, in experience or knowledge and technology, etc.

The proposed method checks the validity of the approximation, testing whether the value of intellectual capital is manifested in the increase of the value of the company.

Also, specific assumptions for the selection of indicators are necessary. On the one hand, the components into which intellectual capital is divided must be indicated. Scholars have already analyzed this problem, and there are different possibilities [3,10,46,65–68].

On the other hand, it is necessary to indicate the aggregation system of these components, to indicate the intellectual capital of the company. Normally, it is assumed that the intellectual capital in the company is the sum of its components [27], but other methodologies use different aggregations [36], while some authors do not aggregate the components [12].

Finally, the available information has associated implicit assumptions. For example, the data must have no measurement errors, once the corresponding indicator has been selected.

The components method requires the user to pinpoint exactly what assets or other monetary manifestations associated with intellectual capital are within the company, as the intellectual capital is intangible and unseen and, therefore, its elements are only detected when used to generate value. In that sense, many models try to be comprehensive, in defining all potential assets of each component of intellectual capital, especially in the scorecard methods [8,10,13,23,53]. However, elements not considered for analysis that may be important always remain, given the difficulty of establishing the full scope of the concept of intellectual capital.

Consequently, each method usually produces a bias. The input-based method generates a certain bias because it identifies the antecedent with the intellectual capital generated by it. The investment approaches are based on the information provided by the profit account, considering expenses related to components of the intellectual capital as investments. Thus, this assumption associates the investment-based approach with methods based on inputs, because the inputs are the expenses. Also, this approach is often associated with component-based methods, because investments are associated with each of the components of intellectual capital. Goebel (2015) [9] criticizes this approach because it considers labor costs to be equated to human capital as an investment, rather than an expense, and the question arises as to whether to add to capital employed.

In parallel, Goebel (2015) [9] criticizes the use of components. On the one hand, it is difficult to estimate the value of each component, as the firm rarely publishes quantitative information on individual components of intellectual capital. Moreover, the interactions are not observable, so in practice, they are omitted, because the measures are focused on individual components [16] when normally the interactions of the components of intellectual capital contribute significantly to the value of intellectual capital [37].

Despite these criticisms, these methods are very often used in the literature of intellectual capital [11,23,25,53]. In particular, one of the most popular methods is the value-added intellectual capital (VAIC) model [27], which suggests VAIC as an indicator of intellectual capital. This method is component-based. The Pulic (2000) [27] model adds additional assumptions that estimate the value generated by intellectual capital highly questionable [9,30,48]. This study uses some of its less questionable elements.

Lev et al. (2016) [51] measure organizational capital through the expenses indicated by the categories of selling, general, and administration. The capital of the organization is described as an efficiency measure for investments in employees, systems, brands, etc. Besides, Nazari and Herremans (2007) [14], among other authors, suggest advertising costs as an indicator of relational capital. Other studies present variations to perform the VAIC model, like the adjustment VAIC [69].

The use of a specific indicator implies an assumption about the meaning of the intellectual capital element associated with this indicator.

Holistic-based market approaches assume that the market incorporates the intellectual capital value beyond the financial statements [1,7]. This approach focuses on the holistic effect of the value of intellectual capital in the value of the company, so that interactions among the components of intellectual capital are captured in the overall value. Intellectual capital investments result in higher revenues generated, with an effect on the value of the company, as argued [70], even if they go beyond financial information.

This approach also has two associated assumptions that have been considered in the work of Stewart (1997) [7].

A3: The intellectual capital of the company is the only factor to increase the value of the company.

A4: The value of the intellectual capital is manifested in the increase in the value of the company.

Consequently, the intellectual capital of the company is the difference between the company value in the market and the book value.

According to these assumptions, in this approach, the objectives are completely different, and the measure of intellectual capital is oriented toward finding indicators, that measure the difference between the company value in the market and the book value of the company. Different indicators suggest different measurement models of intellectual capital. In particular, Goebel (2015) [9] analyzes these indicators and establishes several assumptions about the quality of one or the other.

The consequence of these assumptions is that they identify intellectual capital with the value generated by it and the rest of the sources of capital, becoming a tautology. Moreover, this assumption concludes that all the market value generated is due to intellectual capital, without considering the possible influence of tangible assets as multiplying the value, when interacting with other assets.

The preliminary analysis indicates that the measures used, condition the measured amount of intellectual capital and, consequently, the evaluation of this intellectual capital within the company, and the impact it could have on value creation.

This study aims to measure intellectual capital with public financial data, which establishes indicators for decision making by the manager and, while estimating the efficiency of each of these indicators to have information, for determining how the impact of the use of this indicator affects the results of the company.

The approach combines inputs and outputs, using the advantages of both methods, i.e., formative indicators of the intellectual capital of the company, and the impact that produced these formative indicators, on the consequences of the intellectual capital (reflective indicators). Consequently, the approach solves the common aggregation problem and deals with the criticisms of Goebel (2015) [9] of the input and component methods. In particular, the study uses the expenses as formative indicators, and value-added to the company as a reflective indicator [9,12,24,27].

Summarizing the previous papers, this paper uses the assumptions A1, A2, and A3 to construct an indicator of intellectual capital. Complementarily, the model must include a new assumption (A5) about each of the indicators, that will be specified.

A5: The available financial data on expenses, or investments of intangible assets, summarize the information necessary to evaluate the generation of value due to intellectual capital.

**Fig 1:**
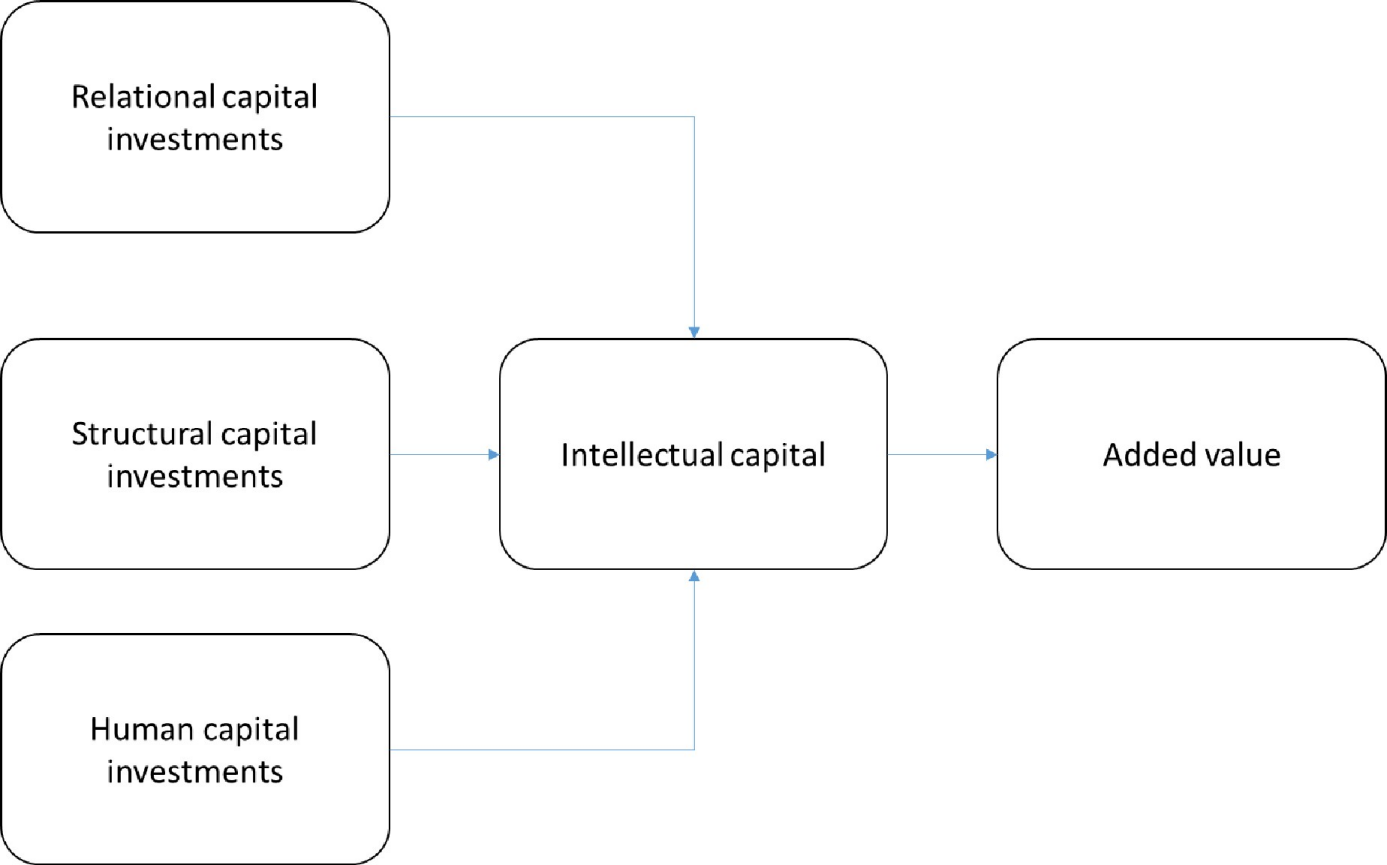
Model estimation of intellectual capital.

According to these assumptions, we can establish a path model, where expenses or investment in each component of intellectual capital generates intellectual capital, and this generates value in the company (see Fig 1).

## 3. Methodology

### 3.1. Data source

The sample of the study population contains annual data from listed European companies, observed in panel data from 2004 to 2015. The data set includes active companies (from January 2004) listed with annual reports, representing all economic sectors and distribution industries. The database has been obtained by combining information collected from the Bureau Van Dijk (Amadeus) and Bloomberg. The constant database of financial indicators that vary various quantitative characteristics specific to intellectual capital and value generation. The database includes annual statistics figures and financial reports.

The information refers to companies located in five European countries: United Kingdom (44 percent), Germany (24 percent), France (25 percent), Spain (5 percent), and Italy (2 percent). The GDP of these countries covers more than 70 percent of the European GDP. The composition of this database represents the European market according to the criteria of the country. We also accurately represent these countries about the industrial structure of the European economy. The Statistical Classification of Economic Activities in the European Community (NACE) was applied, and the following sectors are included in the database: Construction & Real Estate (10%), Manufacturing (27%), Energy & Chemical (5%), Services (12%), Trade & Related Services (10%), Finance & Insurance (12%) and Professional Services (24%). The representative rate for SMEs and large companies in the database is 36 and 64 percent, respectively.

Table 2 outlines the classification of the companies, by industry and country:

**Table 2:** Number of companies by industry and country.

*country*	FRANCE	GERMANY	ITALY	SPAIN	UNITED KINGDOM	*Totals*
*construction and real estate*	45	35	4	19	59	*162*
*manufacturing*	137	134	16	27	153	*467*
*energy and chemical*	9	15	2	8	57	*91*
*services*	70	37	7	9	74	*197*
*trade and related services*	39	46	4	6	62	*157*
*finance and insurance*	23	25	3	3	154	*208*
*professional services*	107	108	8	8	180	*411*
*Totals*	**430**	**400**	**44**	**80**	**739**	*1693*

### 3.2. Measurements of variables

Following previous assumptions, this paper uses costs as an indicator of investments in intellectual capital [11,27,71]. Therefore, personnel costs are an indicator of investment in human capital [68], the costs of R&D are an indicator of structural capital [72], and advertising expenses are an indicator of relational capital [14].

Personnel costs, as an indicator of human capital, have been used previously in the literature, for example, in the VAIC model [27]. This indicator includes the quantity and the quality of human capital, regardless of the contribution of each person, as it may include a few very high salaries. However, it does not discriminate between one aspect and the other, making it difficult to detect which aspect of human capital is more important. In addition, there are aspects of human capital—for example, level of education, values, and attitudes—or some capabilities that are not usually valued in the labor market directly.

Different authors use R&D expenses as an indicator of structural capital [11,73], because it is assumed that the companies investing in R&D, have a structure that promotes a culture of innovation and use of technology, that makes those investments worthwhile, as well as indicating future profitability [74]. However, using spending as the sole indicator of structural capital probably limits the evaluation of this component, because many aspects of this capital, such as culture, technology, and organizational system, are not explicitly included in those costs.

The use of advertising expenses, as an indicator of relational capital [14,73], shows that the company makes an effort to improve its sales and, in that sense, to improve relations with customers. However, other aspects of relational capital, such as the relationship with suppliers, company image, alliances, and cooperation networks, are not directly included. This indicator is assuming that, if the company tries to improve customer relations, then it will be making efforts to improve other aspects of relational capital.

Intangible assets consist of rights that are capable of economic assessment, that is identifiable, nonmonetary, and lack a physical appearance. Besides, they must meet the definition of an asset, that is, there can be control over it, which can be measured reliably, and have the capacity to bring economic benefits.

Amador Fernández and Romano Aparicio (2013) [75] argue that to meet this feature one must verify two conditions: a) the rights are separable and b) they arise from legal or contractual rights. The first condition suggests that they can be sold, transferred, or delivered for exploitation, leasing, or exchange, either individually or together with other related assets or liabilities. The second condition indicates that these aspects arise from legal rights, regardless of whether they are transferable or separable from the company.

Consequently, many of the elements of the intellectual capital of the company cannot be incorporated into this concept because the requirement of identifiability in intangible assets means that, when an asset is not produced and or no payment is explicitly made for such asset, as in the case of goodwill, customer loyalty, or reliance on suppliers, it is not separable.

However, it is possible for intangible assets to be used, as an additional indicator of the stock of intellectual capital, assuming a certain proportionality between identifiable and unidentifiable intangible assets.

Finally, the metric makes use of the value-added, measured by revenue minus current expenses, as an indicator of the value in the company [27], as added value is summarized as the economic value created in the company, from external economic inputs.

The research includes the industry as a control variable, according to the methodology chosen [9].

To eliminate the effect of firm size [76], all variables are introduced divided by the total assets.

### 3.3. Econometric methodology

The research uses the partial least squares technique to test the model (Chin, Marcolin, & Newsted, 1996) [77]. This technique estimates the internal and external relationships in structural equation models (SEM). Solutions based on partial least squares methodology, try to minimize the variance of all dependent variables in terms of causal variables and do not require the assumption of normality of the variables, which is difficult to verify in this case. This technique does not need psychometric validity measures, as the constructs are formative. It is only necessary to analyze the multicollinearity to justify the different indicators, and the R-squared to validate the global model [77].

## 4. Results

The intellectual capital stock is an estimated value and it has been measured by the indicators. The only case of having more than one indicator is intellectual capital stock and, in that case, it is observed that there is no multicollinearity among the different factors, and the impact of each item on the construct is significant.

Table 3 indicates the trajectories between the variables in the model and the significance of these trajectories. It is observed that all impacts are significant. The main effect on intellectual capital is produced by investments in human capital, followed by investments in relational capital. This result shows the importance of investments in intellectual capital components, to increase the intellectual capital of the company, thereby justifying the different investment policies in this asset class. This result shows the validity of assumption AC1, suggesting the validity of the models based on inputs [3,10,46,51,65–68] as potential indicators of intellectual capital.

**Table 3:** Path of the intellectual capital components on added value.

	Original	Mean.Boot	Std.Error	perc.025	perc.975
HC -> IC	0.498	0.564	0.0835	0.4636	0.76
RC -> IC	0.476	0.465	0.0287	0.417	0.515
SC -> IC	0.463	0.406	0.1106	0.0968	0.491
IC -> Perf	0.222	0.316	0.1039	0.1551	0.514
rsq					
	Original	Mean.Boot	Std.Error	perc.025	perc.975
IC	0.9194	0.926	0.0244	0.885	0.982
Perf	0.0491	0.111	0.0705	0.024	0.265

The stock of intellectual capital affects the results, although its impact is not excessive. This result confirms the AC2 assumptions, coinciding with multiple previous works [11,23,25,27,53].

The R-squared is significant in both cases. In essence, the significance of the trajectories confirms the validity of the model and the proposal that was previously presented, making use of this model. Therefore, it can evaluate the intellectual capital existing in each of the companies. The combination of these two results indicates that the model in Fig 1 is valid for the companies analyzed, and is possibly generalizable to other companies, coinciding with [25,46,78,79]. The results confirm that intellectual capital investments generate value.

Next, we analyze the classification of companies, according to the estimated intellectual capital, classified by countries and sectors.

**Fig 2:**
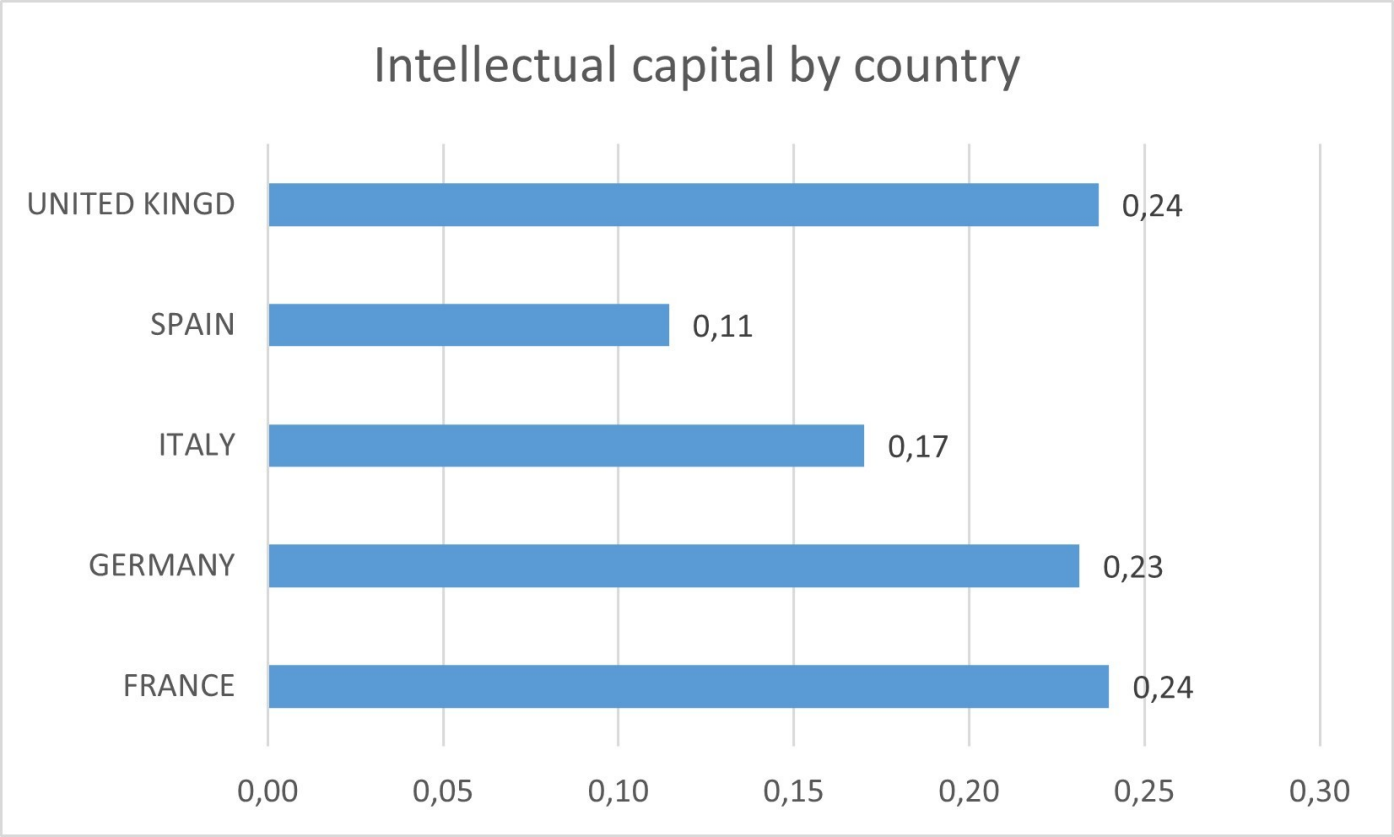
Percentage of intellectual capital stock on total assets by country.

Fig 2 shows the estimate of the value of the intellectual capital stock, concerning the total assets of the company, classified by countries. Companies in Germany, France, and the United Kingdom have a higher percentage of intellectual capital on average than companies in Spain and Italy. The effects of the global financial crisis may have been more noticeable in these last two countries.

**Fig 3:**
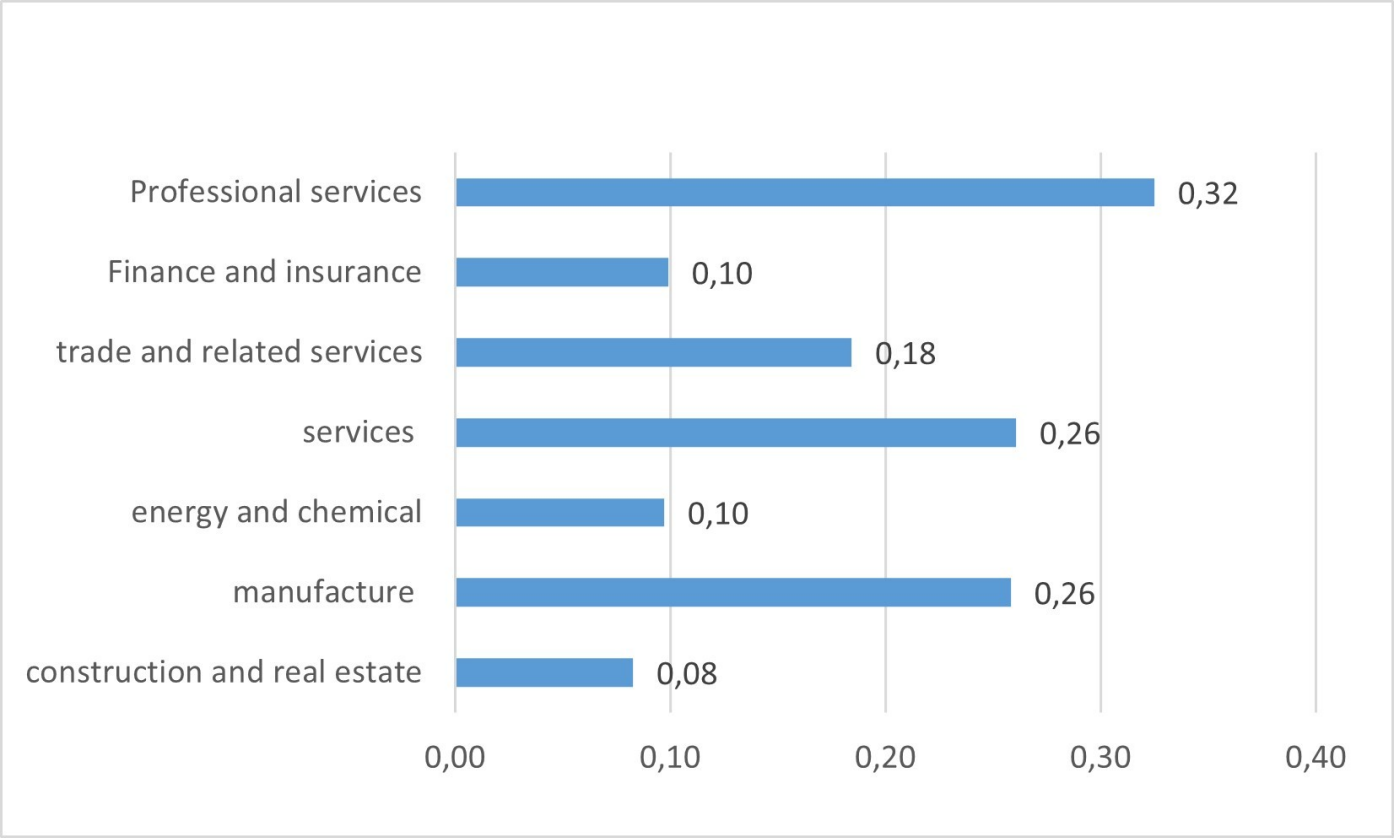
Percentage of intellectual capital stock on total assets by industry.

Similarly, Fig 3 indicates the differences in the stock of intellectual capital, concerning total assets in different industries. It is observed that professional services constitute the highest percentage of intellectual capital, while financial, construction, and energy representations are the least, but this is probably due to the specific financial structure of these industries, specifically the need to finance more investments in tangible assets.

**Fig 4:**
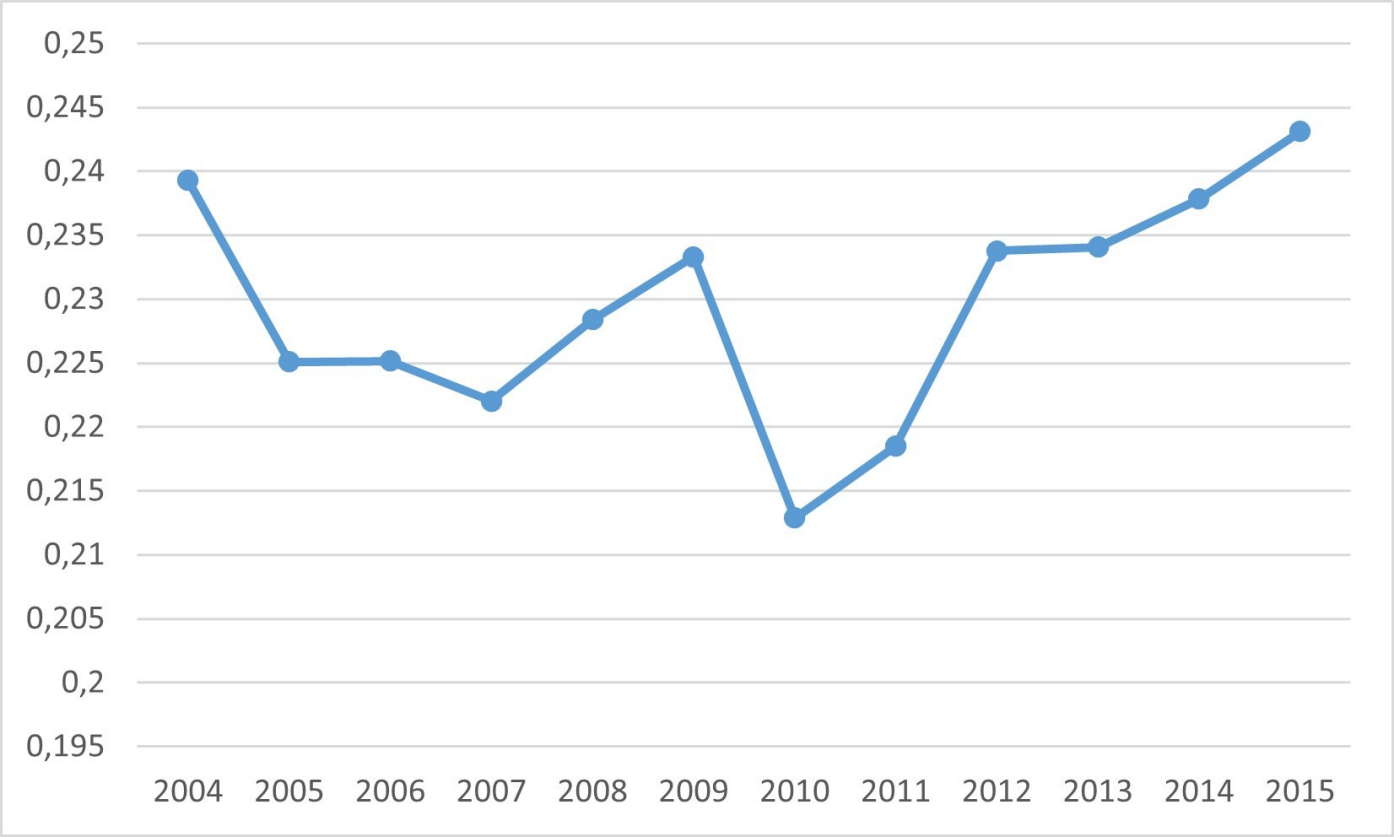
Percentage of intellectual capital stock on total assets by year.

Finally, Fig 4 shows how the intellectual capital, related to total assets, evolves over the years studied. The negative effect of the financial crisis, which reduced the stock of intellectual capital on average, is displayed.

## 5. Discussion

The proposed method combines previous methods, using the advantages of these methods. For example, the method of components has been routinely used, especially since Pulic’s work [27], which has been followed by numerous authors [23,80–82]. Similar to these studies, this method assumes that expenditures are indicators of investments, and similar to other studies in the literature [11,24,73], assumes that the indicators of human capital are personnel expenses, those of structural capital are costs of R&D, and those of relational capital are advertising expenses. The method also presupposes that the added value of the company summarizes the generation of value resulting from intellectual capital [83].

Different authors [9,30] criticize the Pulic method, as the aggregation procedure does not consider the different impact of each component, and the interaction among them. Also, the Pulic method does not discriminate between structural capital and human capital and does not use relational capital [34]. makes use of a corrected version of the Pulic model, considering structural capital to be generated by costs associated with R &D. Other authors [14,73] introduce an indicator of relational capital, but the problem of aggregation is not solved.

The proposed method eliminates the problem of aggregation of the components, common to these previous studies, which usually leads them to directly add the contribution of each component of intellectual capital [23,24,30,73], eliminating its possible different contributions and possible interaction [9].

Sydler et al. (2014) [11] use a model based on the generation of intellectual capital stock from intellectual capital-creating expenses, considering depreciation schemes typical of tangible assets. It uses the expenses considered as investments, making use of the personnel expenses as human capital, of the R&D expenses as structural capital, and the expenses in advertising as relational capital, in a similar way to what is done in the present study. The problem of aggregation arises, assuming that all intangible investments are equivalent so that investments in intellectual capital are obtained as the sum of the three components defined previously.

This study uses the same elements to measure investments in each component of intellectual capital. However, it solves the problem of aggregation through the econometric methodology, because the stock of intellectual capital is defined based on the impact of each of those investments.

Sydler et al. (2014) [11] also consider the generation of the value measured by market value within the model it establishes. The present study simplifies this impact, by making use of added value, although the results are similar to those obtained by [11].

Goebel (2015) [9] summarizes holistic methods, assuming their advantages, due to the limitations of component-based methods. Goebel assumes that the increase in the value of the company is due to intellectual capital. Goebel’s (2015) [9] paper essentially determines which is the best indicator under those premises. The method proposed in the present study meets the Goebel criticisms about input and components methods and assumes that intellectual capital generates value in the company, but, unlike Goebel, does not presuppose that all the value generated is due to intellectual capital; rather, only a part is due to this factor. The results show that only a percentage of total assets is due to intellectual capital on average.

The results have an interest in disclosure. The paper of [84] summarizes the reasons favor and against disclosure; principal reasons against disclosure are direct arguments, like a problem for measurement, and indirect arguments, caused when intangibles information is public. But the fact that this model uses financial data is an advantage, because it resolves problems of measurement, and avoid the problems with public information about intangibles. For example, this work adds information about IC in all European companies analyzed, checking differences by sector and country. But in previous work, about innovation and IC disclosure in European companies, [85], only 29,16% of the companies had disclosure about it.

## 6. Conclusions

This paper presents a structural equation model that tries to measure the intellectual capital of companies using financial data. The model combines component indicators with holistic indicators. This model has not previously been used in measurement methods based on financial data. The possibility to introduce formative and reflective measures allows differentiating the components of intellectual capital from the stock of intellectual capital, an aspect not previously analyzed. Consequently, this method answers a great number of these previous criticisms about the component and holistic methods. To do this, several basic assumptions are set, after analyzing the different measurement models used in previous studies. The indicators obtained are mere indicators; that is, they do not include all the contents of intellectual capital found in the company. However, they suggest which companies have more intellectual capital and how they are used to generate value [9,12,14,16,24,29,31,33,36,86–89].

The paper presents a process to elaborate models of measuring intellectual capital, that is valid with financial data and other types of information. The value of intellectual capital calculated is conditioned by the number and the quality of variables used in the model. In this sense, the criticism of Goebel (2015) [9] about the estimation of the components of intellectual capital is not solved.

This model is applied to a set of European companies, which reinforces the robustness of the work, by referring to a representative set of European public companies. The results show that the indicators established to evaluate the intellectual capital of European companies, meet all the requirements imposed by the previous assumptions. In consequence, they can be accepted as indicators of intellectual capital, justifying models based on inputs or investments, as potential indicators of intellectual capital [11,14,27,48].

The results show, first, that intangible assets can be considered a good indicator of the stock of the intellectual capital of the company. Second, results show that investments in human capital, structural capital, and relational capital increase the intellectual capital of the company [11,14,27,48]. Most important in the generation of intellectual capital is the investment in human capital.

This paper presents several findings that suggest practical implications for two types of agents. On the one hand, for academics, specialists in accounting, and intellectual capital analysts, it is suggested that intangible assets are a potential indicator of the stock of intellectual capital, although, as it is an accounting concept, its use is limited. The findings suggest a measure of intellectual capital that can serve as an indicator to compare companies, and as a way to discover the existing intellectual capital in enterprises, given the need for this to be disclosed [10,37,39,90].

Besides, according to previous literature [11,13,53], the model indicates the impact of intellectual capital on value generation, suggesting an instrument to manage intangible assets [32].

On the other hand, there are several implications for advisors and managers of companies. Investments in intellectual capital are important, but the key investments relate to human resources and human capital. This suggests that training policies, hiring well-trained human resource management, and the overall adequacy of the capabilities of these resources to the company strategy, will be essential value generators in the present circumstances [91–94].

This paper presents some issues that may limit its generalization to other settings. For example, the indicators of intellectual capital are limited by the available information. Future research could use other indicators of interest, which were not introduced, to generalize the results. Finally, the data are limited to the European economy, so the generalization of this model to other economies should be done carefully.

## Supporting information

S1 Annex (DOCX)
