# Correction: Measuring intellectual capital with financial data

**DOI:** 10.1371/journal.pone.0259568

**Published:** 2021-10-29

**Authors:** Carlos M. Jardon, Xavier Martinez-Cobas

The following information is missing from the Funding statement: The authors received funding support from the National Research University Higher School of Economics (NRU HSE). This paper is an output of a research project implemented as part of the Basic Research Program at the National Research University Higher School of Economics. No additional external funding was received for this study.
