# Supplementary material for: Measuring intellectual capital with financial data
Source: PLoS One. 2021 May 3;16(5):e0249989. doi: 10.1371/journal.pone.0249989 (PMC8092649; doi:10.1371/journal.pone.0249989)
Supplement: S1 Annex — (DOCX) [file pone.0249989.s001.docx]

# Supporting Information

## Annex

Table: Description by sector

|  | Obs. | total assets | | added value on assets | | Personnel expenses on assets | | intangible assets on assets | | business expenses on assets | | R & D expenditures on assets | |
| --- | --- | --- | --- | --- | --- | --- | --- | --- | --- | --- | --- | --- | --- |
| Sector |  | Half | Desv. Tip. | Half | Desv. Tip. | Half | Desv. Tip. | Half | Desv. Tip. | Half | Desv. Tip. | Half | Desv. Tip. |
| Building | 1,782 | 995 | 2775 | 0.236 | 0.353 | 0.137 | 0.232 | 0.072 | 0.138 | 0.004 | 0.019 | 0.002 | 0.003 |
| Manufact. | 5,490 | 2252 | 9273 | 0.385 | 0.480 | 0.305 | 2,520 | 0.157 | 0.175 | 0.136 | 0.100 | 0.054 | 0.474 |
| Energy and chemical | 981 | 9014 | 27901 | 0.202 | 0.379 | 0.093 | 0.240 | 0.290 | 1,783 | 0.050 | 0.103 | 0.003 | 0.014 |
| Services | 2,306 | 1501 | 4926 | 0.455 | 1,202 | 0.309 | 0.327 | 0.285 | 0.380 | 0.034 | 0.095 | 0.041 | 0.079 |
| Commerce | 1,850 | 2702 | 8488 | 0399 | 0.294 | 0.261 | 0.197 | 0.142 | 0.162 | 0.037 | 0.043 | 0,005 | 0.028 |
| Finance | 2,375 | 523 | 3519 | 0.163 | 1,420 | 0.114 | 0.464 | 0.120 | 1,524 | 0.030 | 0.377 | 0.003 | 0.031 |
| Serv. prof. | 4,658 | 451 | 2644 | 0.606 | 3,917 | 0.421 | 0.437 | 0.314 | 0.779 | 0.098 | 3,177 | 0.077 | 0.905 |
